# Uncovering the Unusual: A Case of Mixed Connective Tissue Disease With Rare Presentation, Atypical Complications, and Therapeutic Dilemmas

**DOI:** 10.7759/cureus.36298

**Published:** 2023-03-17

**Authors:** Rajashree S Khot, Adarsh Patil, Bharatsing D Rathod, Madan Patidar, Prashant P Joshi

**Affiliations:** 1 General Medicine, All India Institute of Medical Sciences, Nagpur, IND

**Keywords:** cytomegalovirus, chatgpt, cytomegalovirus retinitis, pulmonary tuberculosis, autoimmune haemolytic anaemia, mixed connective tissue disease

## Abstract

Mixed connective tissue disease (MCTD) is an overlap syndrome characterized by features of systemic lupus erythematosus, scleroderma, and polymyositis, along with the presence of the U1RNP antibody. A 46-year-old female patient presented with severe anemia, cough, and breathlessness, and was diagnosed with cold agglutinin disease, a type of autoimmune hemolytic anemia (AIHA). Autoimmune workup revealed MCTD by positive antinuclear and U1RNP antibodies. She had bilateral miliary mottling on X-ray and a tree-in-bud appearance on high-resolution computed tomography of the thorax, which were suggestive of pulmonary tuberculosis. Standard therapy with steroids was not advisable. She was subsequently started on anti-tuberculosis treatment (anti-Koch's therapy), followed by steroid therapy and immunosuppressive therapy after three weeks. The patient responded well to treatment, but after two months, she developed cytomegalovirus (CMV) retinitis. Adult-onset CMV disease may occur as a result of primary infection, reinfection, or activation of a latent infection. Although not directly related, it can occur as an atypical association in the setting of immunosuppressive therapy. Morbidity and mortality are significantly increased in this population secondary to infectious potentiation: immunosuppression causes infections, and infections cause AIHA. The management of MCTD and secondary AIHA and immunosuppression poses a therapeutic challenge.

## Introduction

Mixed connective tissue disease (MCTD) is a rare autoimmune disorder characterized by a combination of clinical features of systemic lupus erythematosus (SLE), systemic sclerosis, and polymyositis. The diagnosis of MCTD is based on the presence of a positive anti-U1RNP antibody, which is a specific serological marker for this disease. Autoimmune hemolytic anemia (AIHA) is a rare manifestation of MCTD and can occur as a result of warm, cold, or mixed autoantibodies production against red blood cells [1]. Here, we present a case of a 46-year-old female who presented with AIHA as an initial presentation of MCTD and developed atypical infections posing management challenges.

## Case presentation

**Figure 1:**
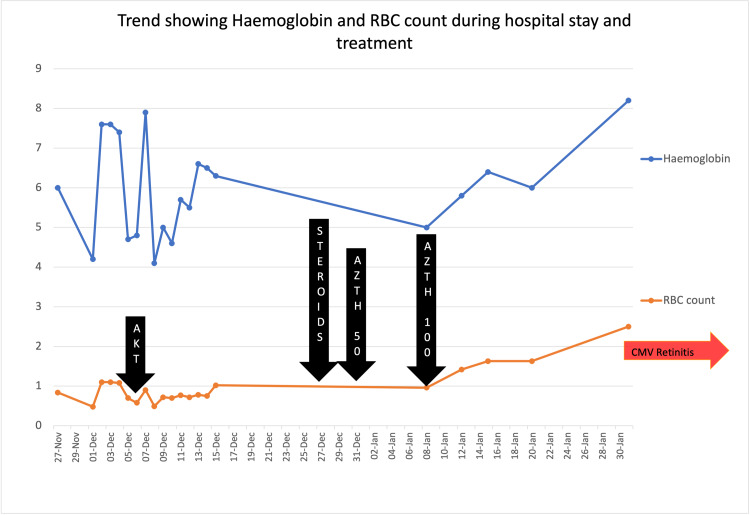
Trend of hemoglobin and RBC count and treatment modalities. AKT: anti-Koch's therapy; AZTH: azathioprine; CMV: cytomegalovirus.

A 46-year-old female was admitted with easy fatigability, breathlessness, and dry cough for 15 days. She gave a history of low-grade fever. She denied hemoptysis, menorrhagia, or bleeding per rectum. There was no history of acrocyanosis, polyarthritis, photosensitivity, edema feet, or oliguria. She had no comorbidities like systemic hypertension, diabetes mellitus, or bronchial asthma. She had genital tuberculosis (TB) in the past for which she had taken antitubercular treatment for nine months. On examination, she had severe pallor, icteric tinge, and bilateral wheezing and rales. On preliminary investigations, her hemoglobin was 6 gm/dl, total leucocyte count (TLC) was 8 x 103, and platelet count was 244 x 103. Her mean corpuscular volume (MCV) was 110.7 femtoliter. She had marked reticulocytosis of 32% with a corrected reticulocyte count of 9.6%. She had indirect hyperbilirubinemia and elevated lactic dehydrogenase (LDH) levels indicating ongoing hemolysis. In two days, her hemoglobin dropped to 4.2 gm/dl and her red blood cell count, total leucocyte count, and platelet count also showed a gradual decline (Figure 1).

**Figure 2:**
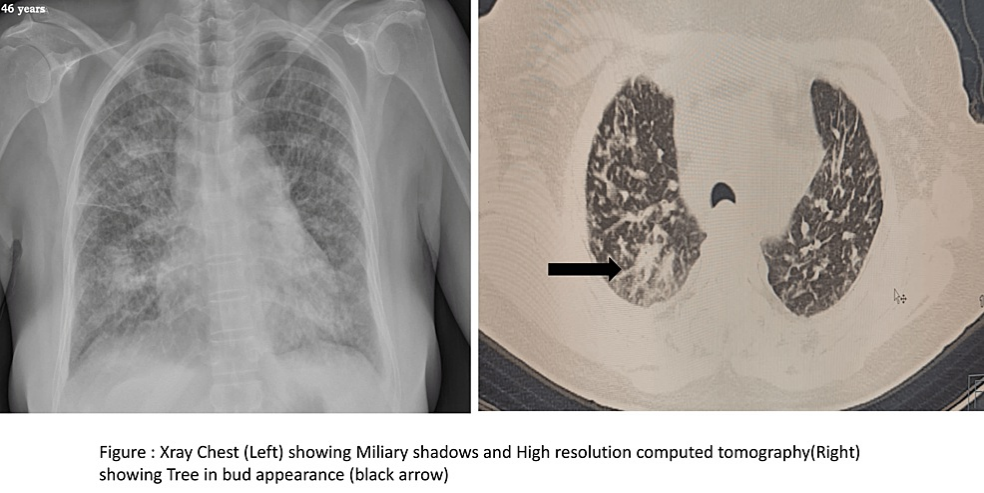
Chest X-ray and thorax HRCT showing radiological features of pulmonary tuberculosis. Tree-in-bud appearance (black arrow). HRCT: high-resolution computed tomography.

**Figure 3:**
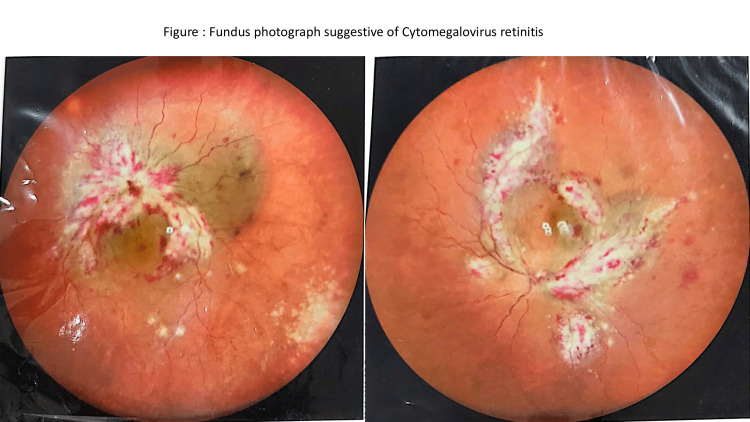
Fundus examination showing bilateral perivascular hemorrhages and exudates as in cytomegalovirus retinitis.

The peripheral blood smear showed microscopic hypochromic RBCs, agglutinated clumps of RBCs, few macrocytes, and few nucleated RBCs. The platelet count was 40,000, and few large platelets were seen. The corrected reticulocyte count was 9.6%. TLC and differential leucocyte counts were normal. Her bone marrow aspiration showed trilineage hematopoiesis with erythroid hyperplasia. On autoimmune workup, antinuclear antibody (ANA) and U1RNP were positive, specific for MCTD. Her direct and indirect Coombs test was positive and she was cold agglutinin positive (IgG/c3). Her chest X-ray showed bilateral miliary shadows, and high-resolution computed tomography (HRCT) showed a tree-in-bud appearance (Figure 2), consistent with TB. Anti-Koch's therapy (AKT) with a four-drug fixed-dose combination was started according to Revised National Tuberculosis Control Program (RNTCP) guidelines. Her symptoms improved but hemolysis persisted. Fifteen days after initiation of AKT, she was given a pulse dose of methylprednisolone for three days followed by oral prednisolone 1 mg/kg. She was also started on oral azathioprine 50 mg daily. Due to continued hemolysis, the dose of azathioprine was escalated to 100 mg daily. Following this, her hemoglobin improved slowly and she started feeling better. Unfortunately, after a month, she developed a blurring of vision in both eyes. Fundus examination showed cytomegalovirus (CMV) retinitis (Figure 3), which was confirmed by serology. Currently, she is receiving intravitreal ganciclovir injections. Her steroid dose has been reduced to half and azathioprine has been discontinued.

## Discussion

Autoimmune disorders can be primary or may be triggered by an infection or a stressful event. MCTD is a rare autoimmune disease that has overlapping features of any of the connective tissue diseases, i.e., SLE, systemic sclerosis, polymyositis, dermatomyositis, and rheumatoid arthritis. As the presentation is variable, diagnosis can be challenging. Hematological manifestations as an initial presentation of MCTD are still rare.

Our patient presented with severe anemia, which was found to be AIHA on investigation. AIHA is a fairly uncommon disorder, with an estimated incidence of one to three cases per 100,000 per year. AIHA is classified by the temperature at which autoantibodies bind optimally to RBCs. Warm antibody AIHA constitutes about 80-90% of adult cases, and 10% of cases have cold agglutinin disease [2].

AIHA can occur due to several reasons, including hereditary, infections, malignancies, or other autoimmune disorders. When we investigated for the cause of AIHA, the patient was found to have MCTD, as her serum ANA and U1RNP were positive in high titers. AIHA has been known to occur in 5-10% of patients with connective tissue diseases. But AIHA as an initial presentation of MCTD is very rare. Our patient did not have any other symptoms of MCTD like myositis or polyarthritis or Raynaud's phenomenon.

Another atypical manifestation in our patient was pulmonary TB. Pulmonary manifestations of MCTD commonly include interstitial lung disease. Hence, we did an HRCT, which showed a tree-in-bud appearance specifically seen in pulmonary TB. She had a history of genital TB, which was treated completely. Now, it is difficult to ascertain whether AIHA due to its propensity for infections caused by TB or the immune response caused by *Mycobacterium tuberculosis* led to the development of IgM-type of antibodies against red blood cells consequently leading to hemolysis. AIHA is an extremely rare occurrence in TB. A total of 21 cases (including adults) that have proven the association of TB with AIHA have been reported so far in the English literature. Most of these were from India and only three cases were reported in children. All cases were direct antiglobulin test (DAT)-positive AIHA. About 46.7% were classified as warm AIHA, 40% as cold AIHA, and 13.3% as a mixed type of AIHA. A case of a 14-year-old child reported by Sheetal et al. was DAT negative but responded to AKT only without steroids [3]. Anurag et al. reported a case of a 25-year-old female who presented with AIHA and simultaneously had pulmonary TB. The patient was managed with blood transfusions and treated with antitubercular treatment [4].

MCTD with AIHA is treated with steroids and immunosuppressants. But we had a therapeutic challenge due to the presence of TB. We deferred this treatment. We initially treated the patient with AKT and supportive blood transfusions. But her anemia did not improve. There was a recurrent drop in hemoglobin levels as hemolysis continued. After three weeks of AKT, we gave her oral steroids. Meanwhile, her leucocyte and platelet count also started to decline. Her pulmonary symptoms were improving slowly. So we added azathioprine at a lower dose and escalated after two weeks. Then her hemolysis decreased and hemoglobin improved to 8.2 gm/dl.

We had another therapeutic challenge as she developed blurring of vision after 15 days and was diagnosed to have CMV retinitis. Infections in AIHA are a known player in the pathogenesis of the autoimmune process. Infections in AIHA can have an impact on the outcome, including morbidity and fatality. The clinical management of infections and prophylactic measures in primary AIHA remain largely unknown, at variance with secondary forms. The only available data are derived mainly from retrospective series and case reports, or more recent clinical trials with novel drugs [5].

The infections in AIHA and MCTD can occur as an effect of treatment with steroids and immunosuppressive agents. The spectrum of infections can vary from bacterial to fungal to viral diseases. Adult-onset CMV disease may occur as a result of primary infection, reinfection, or activation of a latent infection. Although not directly related, it can occur as an atypical association in the setting of immunosuppressive therapy in MCTD [6]. There have been reports of cryptococcal meningitis in AIHA patients receiving prednisolone > 15 mg/day [7].

When safety analysis was done, it was observed that AIHA patients experiencing infections received a mean cumulative dose of corticosteroids of 8.2 kg for a median time of 12 months before the first event [4]. Altogether, data suggest that steroids represent a risk factor for infections, particularly at high doses but also at low and prolonged regimens. Moreover, they are also associated with infections caused by uncommon agents, including fungi and parasites [8].

Immunosuppressive drugs are all associated with an intrinsic infectious risk, generally attributable to bone marrow toxicity. The infectious risk related to cyclosporin A, mycophenolate mofetil, and azathioprine in AIHA is less known since their use in this setting is described mostly as case series or case reports [9].

Primary CMV infection is uncommon in MCTD or SLE. It can occur in patients on immunosuppressive therapy. There has been a single case report of CMV retinitis in an MCTD patient [10]. In our patient, with this new infection, we had to withhold her azathioprine. Further management is still a therapeutic challenge. This case emphasizes that AIHA can be the initial presentation of MCTD and one must carefully evaluate for concurrent infections like pulmonary TB. Infections should be treated first and immunosuppressive therapy should be started gradually. Immunosuppressive therapy can lead to opportunistic infections and physicians should anticipate them.

## Conclusions

This case report highlights an important association between two autoimmune diseases (MCTD and AIHA), an underlying infection (pulmonary TB), and an acquired opportunistic infection (cytomegalovirus retinitis), probably heralded by immunosuppression. All of these represent major therapeutic challenges to the treating physician. AIHA can be the initial presentation of MCTD or a rare complication of TB. Hence, it is important to have a high index of suspicion for the diagnosis of AIHA and its cause. It is also important to monitor and diagnose serious infections when immunosuppressive treatment is used.

## Figures and Tables

**Figure 4:**
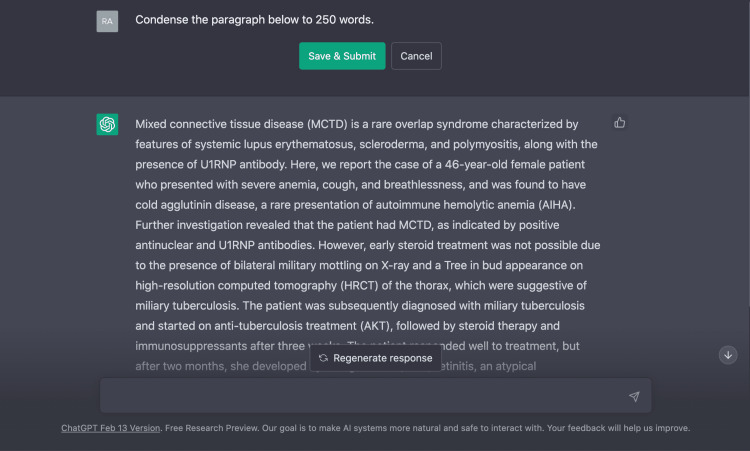
Screenshot of using ChatGPT to limit the word count of the abstract to 250 words.

**Figure 5:**
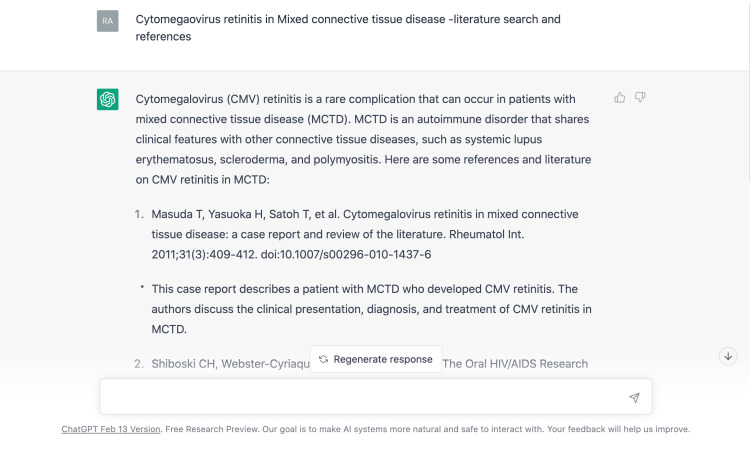
Screenshot of ChatGPT for literature search for CMV retinitis in MCTD. CMV: cytomegalovirus; MCTD: mixed connective tissue disease.

## Appendices

My experience using ChatGPT

I used it for deciding the title for the case report, condensation of the abstract to 250 words (Figure 4), and some specific literature searches (Figure 5).

.
